# The aiming device for cervical distractor pin insertion: a proof-of-concept, feasibility study

**DOI:** 10.1186/s12891-021-04533-w

**Published:** 2021-07-30

**Authors:** Torphong Bunmaprasert, Sittichai Luangkittikong, Menghong Tosinthiti, Supachoke Nivescharoenpisan, Raphi Raphitphan, Nantawit Sugandhavesa, Wongthawat Liawrungrueang, K. Daniel Riew

**Affiliations:** 1 Department of Orthopaedics, Faculty of Medicine, Chiang Mai University, Chiang Mai, 50200 Thailand; 2 Department of Neurological Surgery, Weill Cornell Medicine, New York, NY USA; 3 Department of Orthopedic Surgery At Columbia University, New York, NY USA; 4 The Och Spine Hospital At New York Presbyterian Hospital, New York, NY USA

**Keywords:** Caspar vertebral distraction pin insertion, Anterior cervical surgery, Cervical spine

## Abstract

**Background:**

Restoration of cervical lordosis after anterior discectomy and fusion is a desirable goal. Proper insertion of the vertebral distraction or Caspar pin can assist lordotic restoration by either putting the tips divergently or parallel to the index vertebral endplates. With inexperienced surgeons, the traditional free-hand technique for Caspar pin insertion may require multiple insertion attempts that may compromise the vertebral body and increase radiation exposure during pin localization. Our purpose is to perform a proof-of-concept, feasibility study to evaluate the effectiveness of a pin insertion aiming device for vertebral distraction pin insertion.

**Methods:**

A Smith-Robinson approach and anterior cervical discectomy were performed from C3 to C7 in 10 human cadaveric specimens. Caspar pins were inserted using a novel pin insertion aiming device at C3-4, C4-5, C5-6, and C6-7. The angles between the cervical endplate slope and Caspar pin alignment were measured with lateral cervical imaging.

**Results:**

The average Superior Endplate-to-Caspar Pin angle (SE-CP) and the average Inferior Endplate-to-Caspar Pin angle (IE-CP) were 6.2 ± 2.0° and 6.3 ± 2.2° respectively. For the proximal pins, the SE-CP and the IE-CP were 4.0 ± 1.1°and 5.2 ± 2.4° respectively. For the distal pins, the SE-CP and the IE-CP were 7.7 ± 1.4° and 6.2 ± 2.0° respectively. No cervical endplate violations occurred.

**Conclusion:**

The novel Caspar pin insertion aiming device can control the pin entry points and pin direction with the average SE-CP and average IE-CP of 6.2 ± 2.0° and 6.3 ± 2.2°, respectively. The study shows that the average different angles between the Caspar pin and cervical endplate are less than 7°.

## Background

Anterior cervical discectomy and fusion (ACDF) is a safe and effective operation for degenerative cervical diseases and various disorders. ACDF achieves stable fixation and solid union with reliable clinical results and minimal surgical risks [1]. One of the important steps of ACDF is the distraction of the intervertebral space. This step helps the surgeon to expose the disc space during discectomy, restoring the intervertebral disc height and cervical alignment [2].

**Fig. 1:**
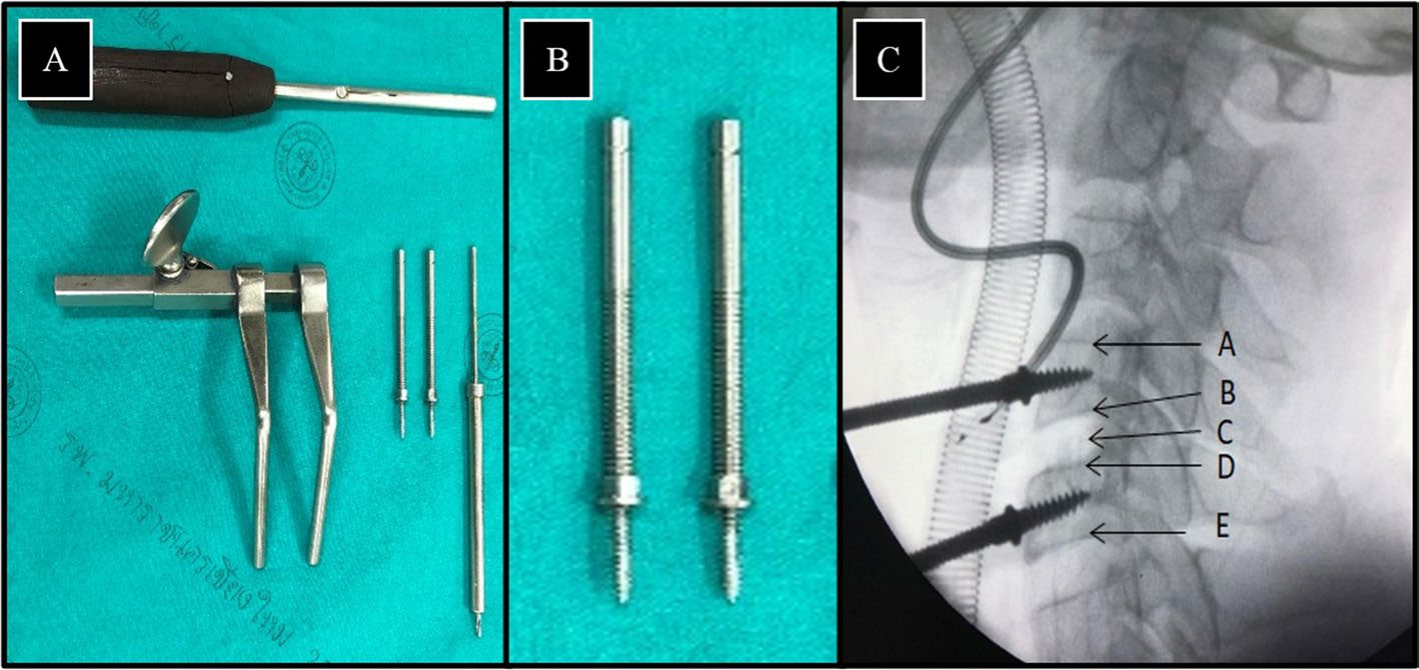
Caspar cervical distractor system (**A**), Caspar cervical pins (**B**), The trajectory and position of Caspar pins at C4-5 in a fluoroscopic lateral view (**C**), (A = superior endplate of C4, B = inferior endplate of C4, C = intervertebral disc of C4-5, D = superior endplate of C5, E = inferior endplate of C5)

The Caspar cervical distractor system (Fig. 1A) is considered a standard tool in performing cervical distraction. Caspar distraction pins (Fig. 1B) may improve disc space visualization and control cervical alignment during direct vertebral distraction (Fig. 1C). The trajectory and position of the Caspar cervical pins affect disc space visualization and working spaces during operation. However, pin malposition may occur even with experienced spine surgeons using a freehand technique, which may result in adverse outcomes [3].

The purpose of this study was to perform a proof-of-concept, feasibility study to evaluate the efficacy of a Caspar pin aiming device for ACDF that was designed to improve the precision of pin placement and minimize radiation exposure during pin insertion.

## Method

### Development and design of Caspar pin aiming device

**Fig. 2:**
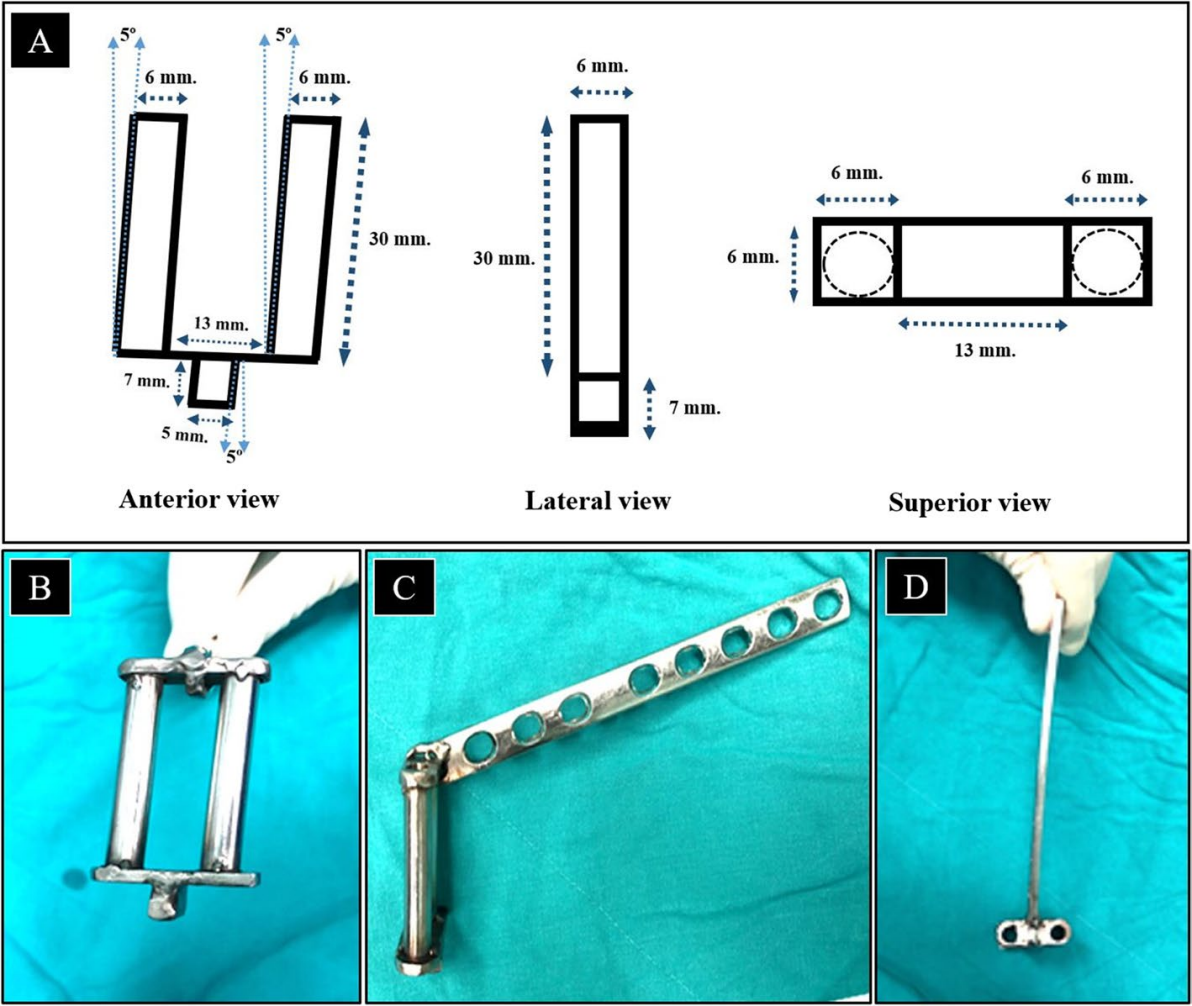
The design of the aiming device (**A**) and actual aiming device anterior view (**B**), lateral view (**C**), and superior view (**D**)

An aiming device was designed and developed (Fig. 2A) with 6 mm diameter sleeves compatible with the diameter of the hexagonal part of the Caspar pin. The sleeves were attached with a 5-degree cranial angulation. The guide was designed with a spacer with a 5-mm width, 7-mm depth and 5° of cranial angulation to fit into the disc space (Fig. 2B, C, D).

### Study subjects

**Fig. 3:**
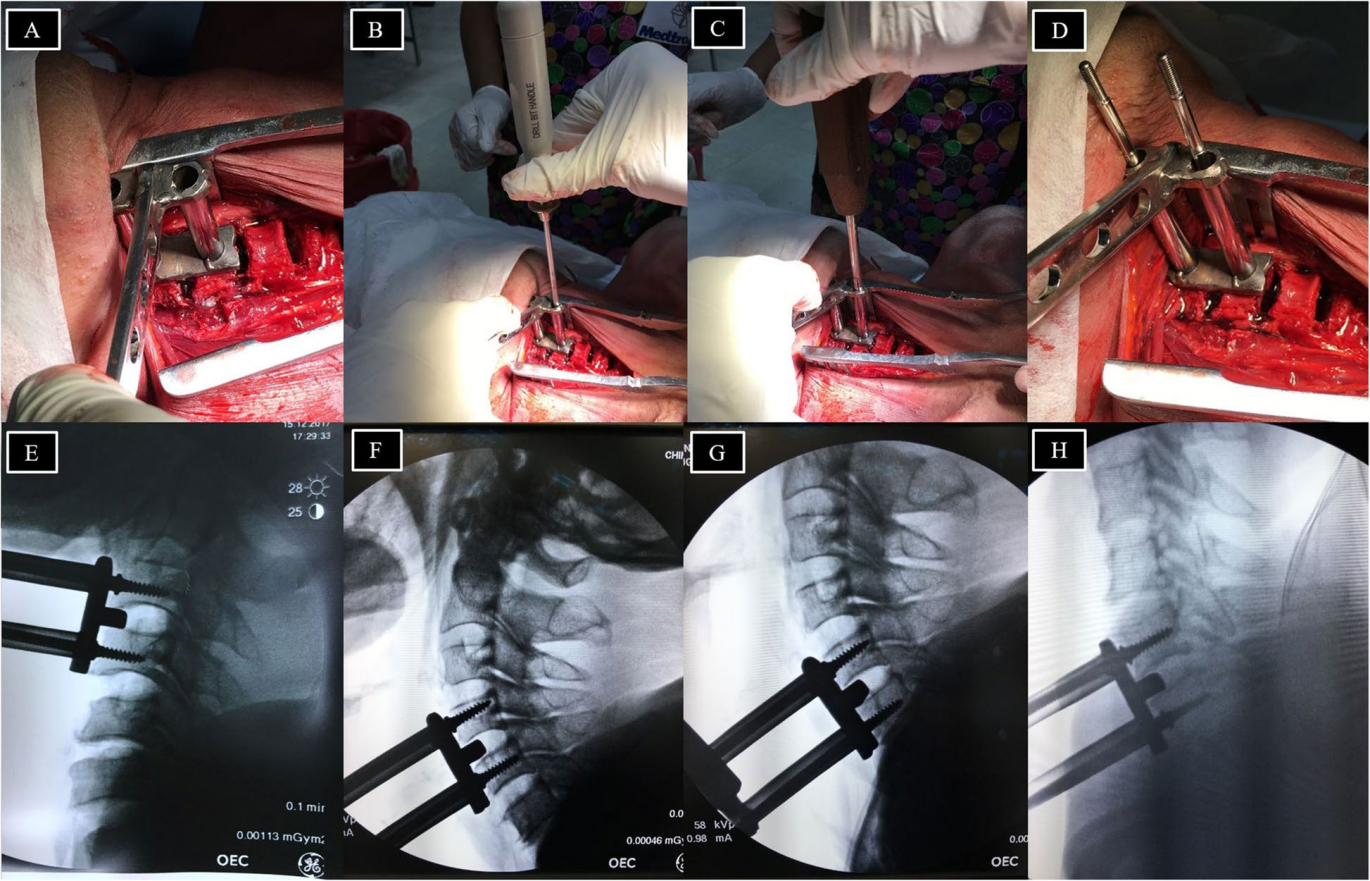
Steps of Caspar pin insertion using the aiming device; Place the aiming device (**A**), Drilling (**B**), First Caspar pin insertion (**C**) and Second Caspar pin insertion (**D**). Lateral fluoroscopic view at C3-4 level (**E**), C4-5 level (**F**), C5-6 level (**G**) and C6-7 level (**H**) of the cervical spine

This study was approved by the University’s Institutional Review Board (IRB number: ORT-2561–05,256). This study was performed on 10 cervical spines from 10 fresh cadavers obtained within 72 h after death. The cadavers came from a donation center of the Faculty of Medicine, Chiang Mai University. The exclusion criteria were a history of cervical spine surgery, severe cervical spine deformity, and severe cervical spine trauma. All dissections were performed by two authors. The cadavers were placed in a supine position with a bar placed transversely under the scapulae to create a slight neck extension. An anterolateral (Smith-Robinson) approach was performed from the right side with a longitudinal incision [4, 5]. Discectomies were sequentially performed at C3-4, C4-5, C5-6, and C6-7 with distraction using the Caspar pin aiming device (Fig. 3A, B, C, D). Lateral imaging of the cervical spine was obtained with fluoroscopy (OEC 9900 Elite, GE Healthcare, Utah, USA) (Fig. 3E, F, G, H).

### Measurements

**Fig. 4:**
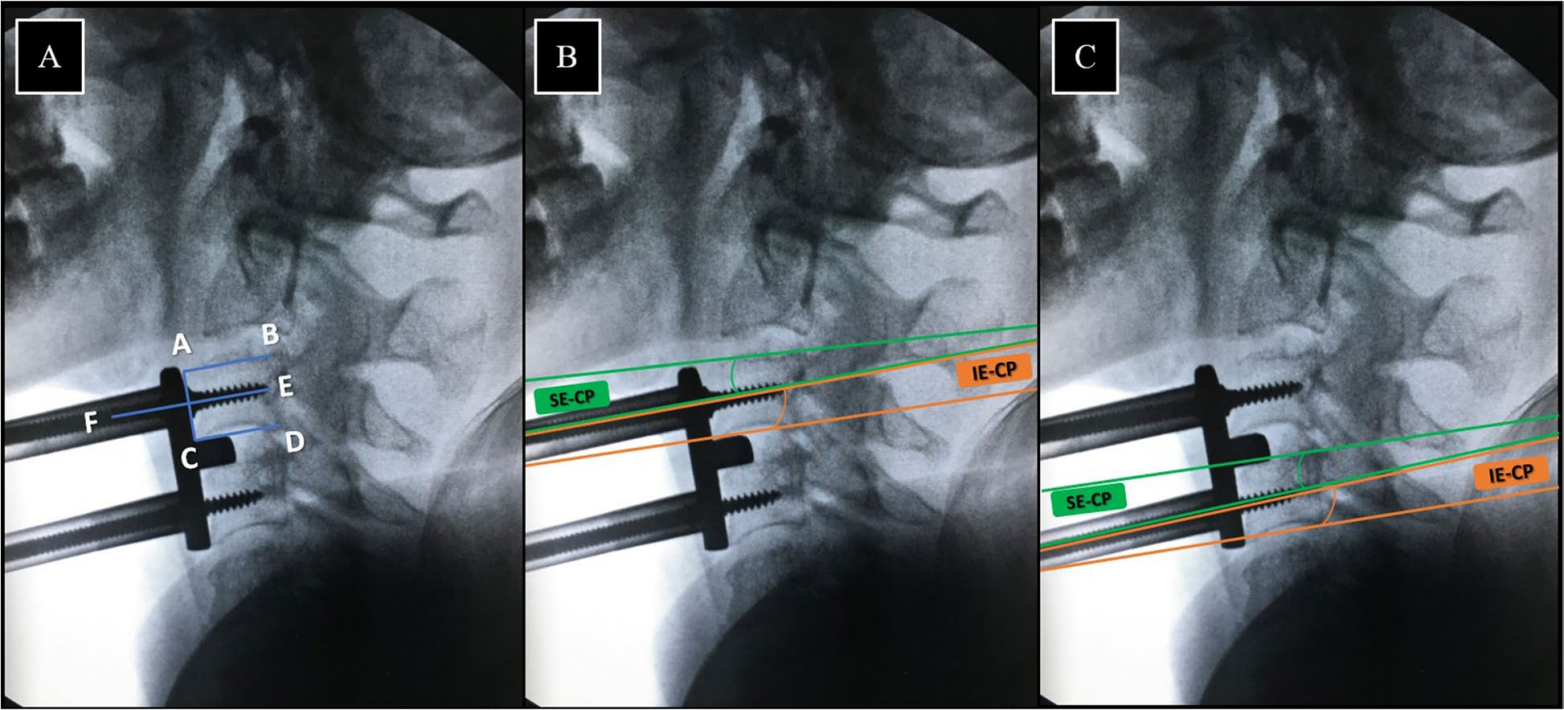
Example of C3-4 after pin insertion; superior endplate slope and inferior endplate slope (**A**), Superior endplate – Caspar pin angle in green line (SE-CP) and Inferior endplate – Caspar pin angle in orange line (IE-CP) (**B**, **C**)

The anatomical landmarks of the vertebral body, including anterosuperior (A), posterosuperior (B), anteroinferior (C), posteroinferior (D) rim and tips of the Caspar pins (E, F) were marked manually using Surgimap Spine. The following parameters were measured: (1) superior cervical endplate slope: the angle formed by AB and AC lines, then subtract that value from 90, (2) inferior cervical endplate slope: angle formed by CD and AC lines, then subtract that value from 90, (3) Superior endplate – Caspar pin angle (SE-CP): the angle formed by AB and EF lines, (4) Inferior endplate – Caspar pin angle (IE-CP): the angle formed by CD and EF lines (Fig. 4).

### Statistical methods

Descriptive statistics (means and standard deviations) were evaluated for quantitative variables. All continuous variables were tested for their normality using the Shapiro–Wilk test. The normal distributed continuous variables were compared using the paired t-test. The statistical significance was set at *P*-value < 0.05. The data were analyzed using IBM SPSS Statistics (version 20, IBM Corporation, Armonk, New York).

## Results

### Cervical endplate slope (superior and inferior endplate slope)

The average age of the cadavers at the time of death was 73.5 years (range 55–88 years). There were five male and five female cadavers. The gender did not influence the cervical endplate slope significantly (*P*-value = 0.06). No significant differences were found between the superior and inferior endplate slopes except C3 (11.7 ± 3.4° vs. 7.4 ± 5.5°, *P*-value = 0.048), suggesting that the endplate slopes were mostly parallel in this study. The average superior endplate slope was 9.6 ± 1.7° (7.4° to 11.7°), and inferior endplate slopes were 6.8 ± 0.9° (6.0° to 8.2°). C3 had largest superior endplate slope (11.7 ± 3.4°), C7 had smallest superior endplate slope (7.4 ± 3.3°), C6 had the largest inferior endplate slope (8.2 ± 3.8°), C7 had smallest inferior endplate slope (6.0 ± 2.9°). The average superior endplate had larger slope compared with inferior endplate (9.6 ± 1.7° vs. 6.8 ± 0.9°, *P* = 0.005) (Table 1).

**Table 1:** Cervical endplate slope (superior and inferior endplate slope)

Level	Superior endplate slope (Mean ± SD)	Inferior endplate slope (Mean ± SD)	*P*-value
C3	11.7 ± 3.4°	7.4 ± 5.5°	0.048*
C4	8.5 ± 1.8°	6.2 ± 3.1°	0.060
C5	9.8 ± 4.1°	6.3 ± 3.4°	0.057
C6	10.6 ± 4.1°	8.2 ± 3.8°	0.200
C7	7.4 ± 3.3°	6.0 ± 2.9°	0.310
Average	9.6 ± 1.7°	6.8 ± 0.9°	0.005*

^*^P-Value < 0.05 that statically significant difference

### SE-CP and IE-CP angle using level vertebral body reference

After Caspar pin insertion with the aiming device, the average Superior endplate – Caspar pin angle (SE-CP) was 6.2 ± 2.0°. The largest of SE-CP was C7 (9.8 ± 3.9°) and the smallest was C4 (4.9 ± 4.0°). The average Inferior endplate – Caspar pin angle (IE-CP) was 6.3 ± 2.2°. The largest of IE-CP was C7 (8.8 ± 3.4°) and the smallest was C5 (4.0 ± 2.7°). At C3, SE-CP had smaller angle than IE-CP (5.6 ± 5.0° vs. 8.7 ± 6.2°, *P* = 0.02). However, no significant difference was found between the average SE-CP and IE-CP (6.2 ± 2.0° vs. 6.3 ± 2.2°, *P* = 0.92) (Table 2).

**Table 2:** SE-CP and IE-CP angle using level vertebral body reference

Level	SE-CP (Mean ± SD)	IE-CP (Mean ± SD)	*P*-value
C3	5.6 ± 5.0°	8.7 ± 6.2°	0.020*
C4	4.9 ± 4.0°	4.6 ± 4.3°	0.720
C5	5.1 ± 3.7°	4.0 ± 2.7°	0.150
C6	5.7 ± 3.4°	5.4 ± 3.8°	0.670
C7	9.8 ± 3.9°	8.8 ± 3.4°	0.230
Average	6.2 ± 2.0°	6.3 ± 2.2°	0.920

^*^P-Value < 0.05 that statically significant difference

### SE-CP and IE-CP angle using proximal pin reference

Focusing on the proximal pin insertion with the aiming device, the average SE-CP was 4.0 ± 1.1°. The largest SE-CP was at C3 (5.6 ± 5.0°) and the smallest was C5 (3.0 ± 2.2°). The average IE-CP was 5.2 ± 2.4°. The largest IE-CP was at C3 (8.7 ± 6.2°) and the smallest was at C5 (3.4 ± 1.9°). No significant difference was found between the SE-CP and IE-CP except at C3 (5.6 ± 5.0° vs. 8.7 ± 6.2°, *P* = 0.02) (Table 3).

**Table 3:** SE-CP and IE-CP angle using proximal pin reference

Level	SE-CP (Mean ± SD)	IE-CP (Mean ± SD)	*P*-value
C3	5.6 ± 5.0°	8.7 ± 6.2°	0.020*
C4	3.4 ± 3.3°	4.5 ± 4.9°	0.280
C5	3.0 ± 2.2°	3.4 ± 1.9°	0.760
C6	4.0 ± 3.8°	4.2 ± 5.0°	0.860
Average	4.0 ± 1.1°	5.2 ± 2.4°	0.170

^*^P-Value < 0.05 that statically significant difference

### SE-CP and IE-CP angle using distal pin reference

In the distal pin reference, the average SE-CP was 7.7 ± 1.4°. The largest SE-CP was at C7 (9.8 ± 3.9°) and the smallest was at C4 (6.5 ± 4.3°). The average IE-CP was 6.2 ± 2.0°. The largest IE-CP was at C7 (8.8 ± 3.4°) and the smallest was at C5 (4.6 ± 3.2°). No significant difference was found between the SE-CP and IE-CP except at C5 (7.1 ± 3.8° vs. 4.6 ± 3.2°, *P* = 0.02) (Table 4).

**Table 4:** SE-CP and IE-CP angle using distal pin reference

Level	SE-CP (Mean ± SD)	IE-CP (Mean ± SD)	*P*-value
C4	6.5 ± 4.3°	4.8 ± 3.8°	0.220
C5	7.1 ± 3.8°	4.6 ± 3.2°	0.020*
C6	7.4 ± 1.9°	6.6 ± 1.8°	0.120
C7	9.8 ± 3.9°	8.8 ± 3.4°	0.230
Average	7.7 ± 1.4°	6.2 ± 2.0°	0.260

^*^P-Value < 0.05 that statically significant difference

## Discussion

One of the key goals of ACDF is the restoration of the intervertebral disc height with vertebral distraction and graft insertion, which can indirectly decompress the neural foramina, as well as the spinal canal [6, 7]. The Caspar distractor system is widely used to achieve such distraction, as it improves the exposure of the intervertebral space, making it easier and safer to perform the decompression [2, 8].

The technique of pin insertion is very important for visualization and maintaining the working space during the operation. Malposition, such as the pin being placed too close to the operative disc, can result in interfering with the endplate preparation, while those placed too distally can cut thru adjacent endplates, especially in osteoporotic patients. In the coronal plane, the entry points of the Caspar pins should be in the midline. Off-center pins can create vertebral rotation and scoliosis. If the pins are both placed off to one side, distraction can cause asymmetrical endplate preparation. Moreover, inserting the pins off to one side can compromise plate fixation if it is in close proximity to where the screw needs to be inserted into. For the pin direction, the cranial angulation of the pin should be parallel to the disc space in the sagittal plane. Previous studies had no consensus about the appropriate trajectory for placement of Caspar pins. Only one study suggested that the cranial pins be inserted at the upper third of the vertebral body to be fused and the caudal pins be placed at the lower third of the vertebral body under fluoroscopic guidance [9]. For an ACDF operation, the superior pin is ideally approximately 7 to 10 mm from the upper level’s inferior endplate because of the concavity of that endplate. The inferior pin can be placed approximately 5 mm below the lower level’s superior endplate [8] (Fig. 1C). However, when performing a total disc replacement operation (TDR), the Caspar pins must be placed as far from the operative level as possible without violating the adjacent discs. In a previous study in an Asian population, Chen et al. reported that the average cranial angulation of the superior endplates ranged from 4.5 to 9.0° and the average cranial angulation of inferior endplates were between 4.5 to 7.5° [10]. Yukawa et al. reported that the minimal disc height of the cervical vertebra was 5.8 ± 1.3 mm [11].

For most patients with normal anatomy, the ideal position of the Caspar pin is in the center of the anterior vertebral body in the coronal plane and parallel to the vertebral endplates [12]. Caspar pins are typically inserted by free-hand technique with or without intraoperative imaging [2]. However, this technique can result in nearly perfect pin placement or sometimes require multiple attempts of pin insertion that can compromise the vertebral body and necessitate increased radiation exposure.

Our study shows that the average superior and inferior endplate slopes are different from the previous study by Chen et al. (superior endplate slope; 7.4° to 11.7° vs. 4.5° to 9.0°, inferior endplate slope; 6.0° to 8.2° vs. 4.5° to 7.5°). One reason may be that the age (at the time of death) in this study is older compared to the previous study (73.5 years, range 55–88 years vs. 41 years, range 25–51 years) [10]. The purpose of our study is to develop and test a novel aiming device prototype to control the pin entry points as well as direction. The pin entry point is 7 mm. from each side of the vertebral endplate. The center in the coronal plane is found by identifying the uncinate process bilaterally and placing the aiming device in the middle. The aiming sleeves then guide the pin direction in a cephalad inclination identical to the cervical endplates. The study shows the average SE-CP was 6.2 ± 2.0° and the average IE-CP was 6.3 ± 2.2°. In the present study, we do not observe any cervical endplate violation in any of the Caspar pin insertions from C3 to C7.

The study has some limitations. First, this is only a cadaveric study designed to be a proof-of-concept feasibility trial [13]. Another shortcoming is the relatively small number of cadavers utilized. But these were Asian subjects and their vertebral sizes are smaller than other races [14]. Therefore, if it worked in these relatively smaller necks, it should work in larger subjects. Third, it is still unclear if the device would be effective in highly kyphotic individuals. It may be necessary to place pins in such cases without the guide using the traditional free-hand technique. Finally, the device may not be applicable for all surgeons, especially ones prefer to insert the pins before performing a partial discectomy. Highly experienced surgeons may find our guide to be unnecessary. Therefore, the guide is not for everyone. But if a surgeon finds reliable pin placement to be somewhat troublesome, they may find that our guide provides some benefits. We believe that it will ultimately be a personal preference issue.

The ideal patients who are suitable for this device may be less kyphotic sagittal alignment and nearly-preserved disc height. We recommend checking a lateral image once the device is in place, prior to inserting the pins, which should help to ensure that the pins will not violate the adjacent endplates in small individuals.

### Clinical application

**Fig. 5:**
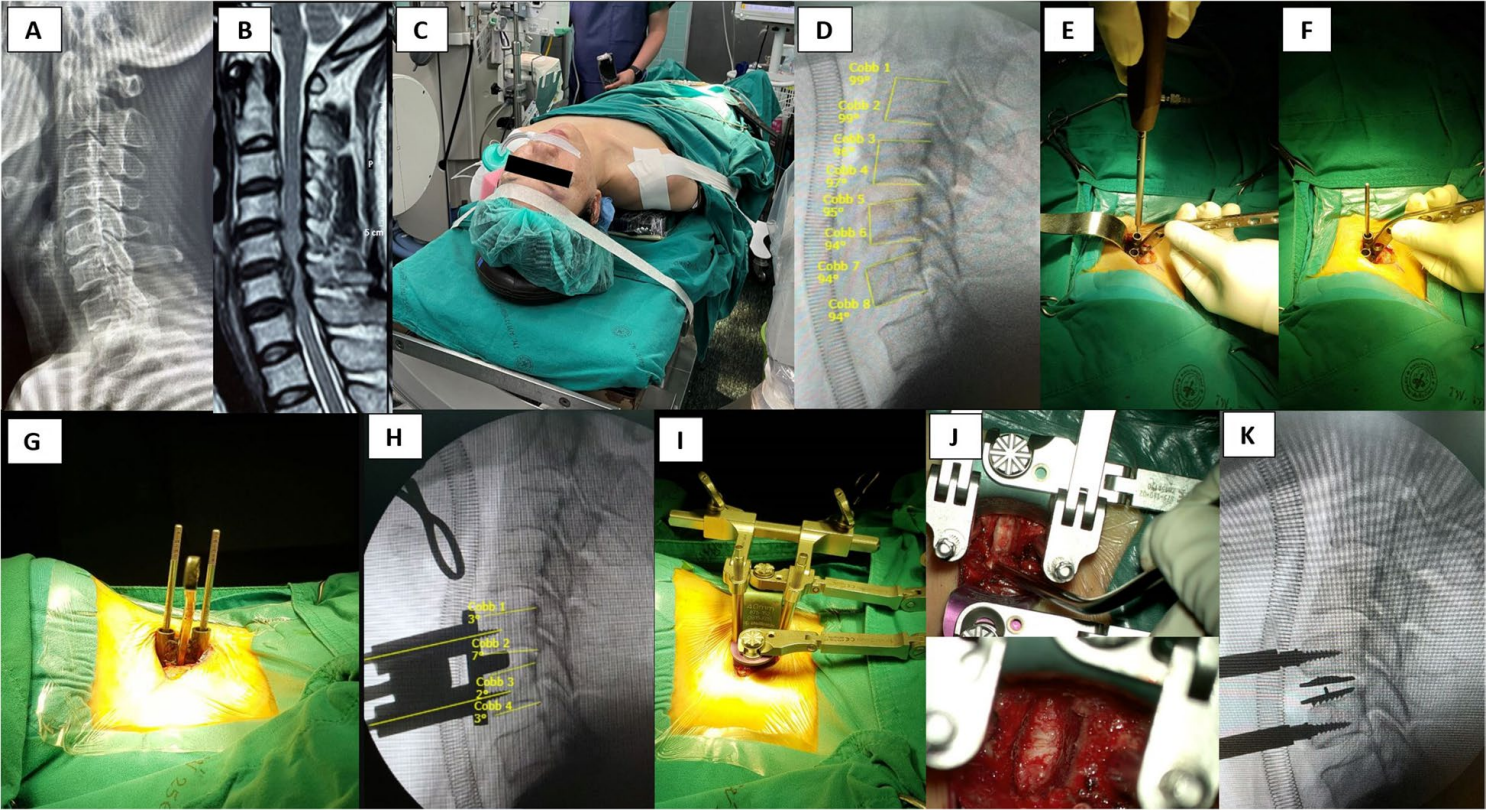
Patient 1 was performed Single-level C-TDR C5-6. Preoperative lateral X-ray (**A**), Sagittal MRI (**B**), Patient positioning (**C**), Intraoperative measurement of C5-C6 superior and inferior endplate angle (**D**), First Caspar pin placement using the aiming device (**E**, **F**), Second Caspar pin placement (**G**), C5-6 Caspar pin placement measuring SE-CP & IE-CP angles (**H**), C5-6 Caspar cervical distractor placement (**I**), Caspar cervical distractor system creating the working space (**J**), Intraoperative lateral fluoroscopic view (**K**)

**Fig. 6:**
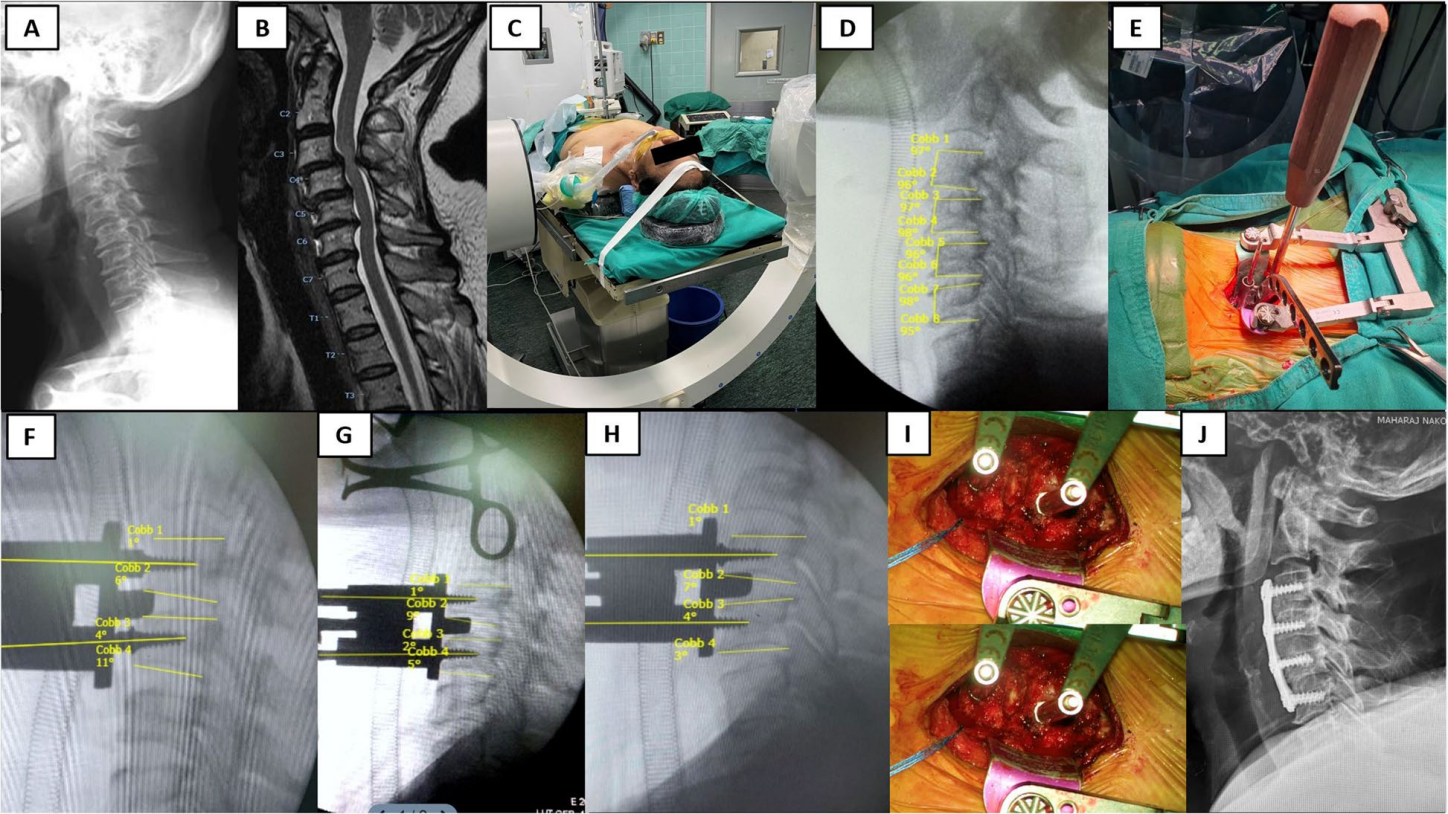
Patient 2 was performed Multilevel ACDF C3-4 C4-5 C5-6. Preoperative lateral X-ray (**A**), Sagittal MRI (**B**), Patient positioning (**C**), Intraoperative measurement of C3-C4-C5-C6 superior and inferior endplate angle (**D**), Caspar pin placement using the aiming device (**E**), C3-4 Caspar pin placement (**F**), C4-5 Caspar pin placement (**G**), C5-6 Caspar pin placement (**H**), Caspar cervical distractor system creating the working spaces (**I**), Postoperative lateral X-ray (**J**)

This study was only a cadaveric study. However, the aiming device was utilized and tested by our current spine fellow and orthopaedic resident to apply in two patients. Patient 1 was a 52-year-old female with cervical disc herniation performing single-level C-TDR C5-6 (Fig. 5). Patient 2 was a 61-year-old female with three-level cervical spondylotic myeloradiculopathy (C3-4, C4-5, C5-6) performing ACDF (Fig. 6). These parameters were collected and analysed as Table 5. The aiming device works well at the lower-level subaxial vertebrae. It is more suitable for one-level and two-level disc diseases. The flat, straight plate portion of the aiming device works better if doing the multi-level disc diseases with segment-by-segment insertion, not depend on the previous adjacent pin that has been placed before. That may make another attempt for pin insertion. In markedly-narrow disc space, it is recommended for the surgeon to remove at least the anterior -half of the disc before insertion the aiming device spacer. Placing the pin at the upper segment (i.e. C3-4 disc), C3 is difficult to place the aiming device because the direction may be obstructed by the mandible. According to the angle of pin insertion, SE-CP and IE-CP angles of Patient 1 are related to the findings from the cadaveric study (The study shows that the average different angles between the Caspar pin and cervical endplate are less than 7°). However, we find that IE-CP angle of Patient 2 at C4 vertebra is excess than 7 degrees. That may cause from twice attempts for pin insertion. The authors believe that a clinical study is important for the next stage.

**Table 5:** The aiming device was utilized and tested by spine fellow and orthopaedic resident to apply in two patients

No.	Parameters		Patient 1				Patient 2			
			C3	C4	C5	C6	C3	C4	C5	C6
1.	No of attempts need (times)		-	-	1	1	2	2	2	1
2.	Average duration for pin insertion (minutes)		-	-	3.5	3.5	5.0	3.5	3.5	4.0
3.	Angle of pin insertion (degrees)	Superior Endplate Slope	-	-	5.0	4.0	7.0	7.0	6.0	8.0
		Inferior Endplate Slope	-	-	4.0	4.0	6.0	8.0	6.0	5.0
		SE-CP	-	-	3.0	2.0	1.0	2.5	1.5	4.0
		IE-CP	-	-	7.0	3.0	6.0	9.0	6.0	3.0
4.	Radiation exposure		Intraoperative fluoroscopy did not use to guide the aiming device and insert the Caspar pins in all levels, just used for checking a lateral image once the aiming device was in place.							

## Conclusion

The Caspar vertebral distraction pin aiming device in the present study can provide a simple, reliable and reproducible method for Caspar pin insertion. We found that the average angles between the Caspar pin and cervical endplate are less than 7°.

## Data Availability

The datasets used and/or analysed during the current study are available from the corresponding author on reasonable request.
